# ZifBASE: a database of zinc finger proteins and associated resources

**DOI:** 10.1186/1471-2164-10-421

**Published:** 2009-09-09

**Authors:** Mannu Jayakanthan, Jayaraman Muthukumaran, Sanniyasi Chandrasekar, Konika Chawla, Ankita Punetha, Durai Sundar

**Affiliations:** 1 Centre of Excellence in Bioinformatics, School of Life Sciences, Pondicherry University, Pondicherry 605014, India; 2 Department of Biochemical Engineering and Biotechnology, Indian Institute of Technology (IIT) Delhi, Hauz Khas, New Delhi 110016, India

## Abstract

**Background:**

Information on the occurrence of zinc finger protein motifs in genomes is crucial to the developing field of molecular genome engineering. The knowledge of their target DNA-binding sequences is vital to develop chimeric proteins for targeted genome engineering and site-specific gene correction. There is a need to develop a computational resource of zinc finger proteins (ZFP) to identify the potential binding sites and its location, which reduce the time of *in vivo *task, and overcome the difficulties in selecting the specific type of zinc finger protein and the target site in the DNA sequence.

**Description:**

ZifBASE provides an extensive collection of various natural and engineered ZFP. It uses standard names and a genetic and structural classification scheme to present data retrieved from UniProtKB, GenBank, Protein Data Bank, ModBase, Protein Model Portal and the literature. It also incorporates specialized features of ZFP including finger sequences and positions, number of fingers, physiochemical properties, classes, framework, PubMed citations with links to experimental structures (PDB, if available) and modeled structures of natural zinc finger proteins. ZifBASE provides information on zinc finger proteins (both natural and engineered ones), the number of finger units in each of the zinc finger proteins (with multiple fingers), the synergy between the adjacent fingers and their positions. Additionally, it gives the individual finger sequence and their target DNA site to which it binds for better and clear understanding on the interactions of adjacent fingers. The current version of ZifBASE contains 139 entries of which 89 are engineered ZFPs, containing 3-7F totaling to 296 fingers. There are 50 natural zinc finger protein entries ranging from 2-13F, totaling to 307 fingers. It has sequences and structures from literature, Protein Data Bank, ModBase and Protein Model Portal. The interface is cross linked to other public databases like UniprotKB, PDB, ModBase and Protein Model Portal and PubMed for making it more informative.

**Conclusion:**

A database is established to maintain the information of the sequence features, including the class, framework, number of fingers, residues, position, recognition site and physio-chemical properties (molecular weight, isoelectric point) of both natural and engineered zinc finger proteins and dissociation constant of few. ZifBASE can provide more effective and efficient way of accessing the zinc finger protein sequences and their target binding sites with the links to their three-dimensional structures. All the data and functions are available at the advanced web-based search interface .

## Background

The capability to manipulate and alter the various organisms has been one of the supreme achievements of modern molecular biologists. Custom designed zinc finger proteins (ZFPs) provide a powerful platform technology since other functional domains like non-specific *Fok *I cleavage domain (N), transcription activator domain (A), transcription repressor domain (R) and methylase (M) can be fused to the ZFPs to form zinc finger nucleases (ZFNs), zinc finger transcription activators (ZFA), zinc finger transcription repressors (ZFR) and zinc finger methylases (ZFM) respectively [1].

ZifBASE is a comprehensive resource to obtain detailed information on naturally occurring and engineered ZFPs, hyperlinks to the three dimensional structures for naturally occurring ZFPs, finger positions, number of fingers, physiochemical properties and all classes (namely C2H2, CXXC, PHD etc.) and framework (namely Zif268, Sp1C, Sp1C-Zif268, TF(1-4)-Zif268), with PubMed citations. The existing resources [2-7] for ZFPs are databases of only individual modules and engineered arrays with their binding sites. ZifBASE organizes information on the zinc fingers of *Bos taurus*, *Danio rerio*, *Drosophila melanogaster*, *Fugu rubripes*, *Gallus gallus*, *Homo sapiens*, *Mesocricetus auratus*, *Mus musculus*, *Rattus norvegicus*, *Trichoderma reesei *and *Xenopus laevis*. All the naturally occurring ZFPs have been linked to UniProtKB, PDB, Modbase and Protein Model Portal to enhance its utility. The major merits of ZifBASE are (i) combination of datasets for both engineered and naturally occurring zinc finger proteins, the number of finger units in each of the zinc finger proteins (with multiple fingers) and the synergy between the adjacent fingers and their positions. (ii) not restricted to any particular organism (iii) the search interface offers finding of unique patterns/potential ZFP binding sites with user-given inputs to find in combination of forward and complementary strand (iv) the details on class of natural ZFP and framework of engineered ZFP (v) the full length protein sequence of engineered constructs and naturally occurring ZFPs (vi) physiochemical properties which are important to understand the DNA-binding affinity of the ZFPs and (vii) link to three dimensional structures [experimental (if available) and models] of natural ZFPs. Three dimensional structural studies of ZFPs have given insights on the ZFP-DNA- binding interface and it can be compared with respect to a 'canonical binding model' where each finger interacts with DNA in an anti-parallel mode. The structures can also be analyzed to identify the α-helical regions in each finger that fits into the major groove of the duplex DNA with base contacts. This provides clear evidence for the protein's ability to bind specifically to a DNA sequence. ZifBASE will be very useful to researchers working in protein engineering of DNA recognition motifs, creating site-specific artificial zinc finger-transcription factors/repressors and zinc finger nucleases to target and achieve highly efficient and permanent genome modification [1].

## Construction and content

### Construction

**Figure 1:**
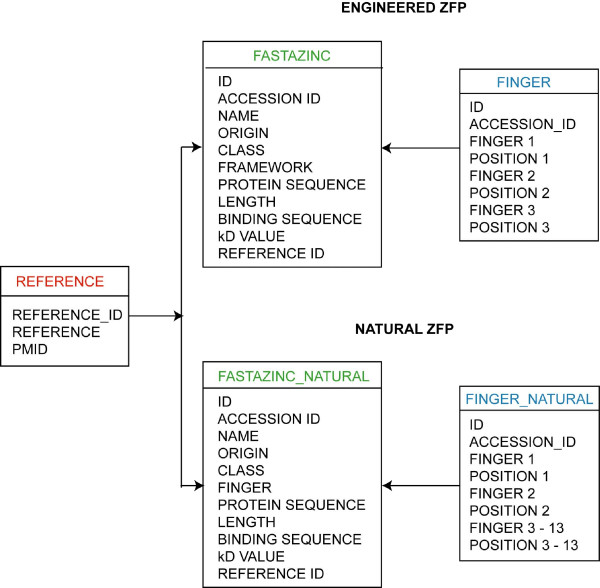
**Entity relationship diagram of ZifBASE**. A layout of the ZifBASE database with various linked tables is shown here. The tables in the database are linked by special constraints in MySQL, namely foreign key.

ZifBASE, a web based platform independent, manually curated database is currently running on Apache web server [8] with the application program Hypertext preprocessor (PHP) [9]. The database tables are stored in MySQL relational database [10], which affords the facility to relate two or more tables in database. Major tables include fastazinc, fastazinc_natural, finger, finger_natural and reference as shown in Figure 1. Unique key was used to maintain the non-redundancy records in ZifBASE database.

### Content

#### Data sources - Engineered and natural ZFP datasets

The natural and engineered ZFPs were collected from various resources such as PubMed [11], UniProtKB [12,13], PDB [14], ModBase [15] and Protein Model Portal [16]. Of the different types of DNA-binding motifs that exist, C2H2 zinc finger proteins are the abundant class of DNA-binding domains observed in human transcription factors. They are found in 2% of all human genes and have proven to be the most versatile of all DNA-binding proteins. Each finger recognizes and binds to three base pair sequence of DNA and three such finger can be assembled together to bind a longer 9 base-pair sequence. The modular structure of ZFP makes it ideal for design or engineering to bind to specific target genome sequence. The naturally occurring zinc finger proteins commonly contain a sequence of the type (Tyr, Phe)-X-Cys-X_2-4_-Cys-X_3_-Phe-X_5_-Leu-X_2_-His-X_3-5_-His, where X represents relatively non-conserved amino acids [17]. Hence, ZifBASE has entries of natural zinc fingers, which have more than 12 residues and lay between the second CYS and the first HIS in finger sequence. But in case of engineered ZFPs, residues at seven positions of α-helix that makes most of the base contacts were considered. Key amino acid residues at positions -1, +1, +2, +3, +4, +5 and +6 relative to the start of the α-helix of ZF motifs contribute to the sequence-specific interactions with the DNA site. The dataset of naturally occurring ZFPs has been organized based on the organism, size (2-13 fingers) and class/type of fingers (C_2_H_2_, CXXC and PHD). The collected data were validated for manual errors, error in data types, redundancy and finally stored in database using SQLyog - MySQL GUI tool. The curated entries of ZifBASE are shown in Tables 1, 2 and 3.

**Table 1:** Details of naturally occurring zinc finger proteins in Zif-BASE

**Source organism**	**Type/Class**	**Amount of ZFPs**	**Number of fingers**
*Bos taurus*	C2H2	2	3, 4
*Bos taurus*	CXXC and PHD	1	2
*Danio rerio*	C2H2	3	3, 4, 6
*Drosophila melanogaster*	C2H2	2	4
*Gallus gallus*	C2H2	3	3, 6, 11
*Fugu rubripes*	C2H2	1	6
*Homo sapiens*	C2H2	16	3, 4, 5, 6, 8, 9, 10, 11, 13
*Homo sapiens*	CXXC and PHD	1	2
*Homo sapiens*	CXXC	1	3
*Mesocricetus auratus*	C2H2	1	4
*Mus musculus*	C2H2	12	3, 4, 5, 6, 7, 11, 13
*Mus musculus*	CXXC and PHD	1	2
*Rattus norvegicus*	C2H2	2	3, 11
*Trichoderma reesei*	C2H2	1	3
*Xenopus laevis*	C2H2	3	6, 11

**Table 2:** Details of engineered zinc finger proteins in Zif-BASE

**Number/size of fingers (F)**	**Framework**	**Number of ZFPs**
3 × 2F	Zif268	1
	Sp1C	-
	Sp1	-
	Sp1C-Zif268	-
	TF(1-4)-Zif268	-
		
3F	Zif268	2
	Sp1C	4
	Sp1	73
	Sp1C-Zif268	-
	TF(1-4)-Zif268	-
		
2 × 3F	Zif268	3
	Sp1C	1
	Sp1	-
	Sp1C-Zif268	3
	TF(1-4)-Zif268	-
		
4F	Zif268	1
	Sp1C	-
	Sp1	-
	Sp1C-Zif268	-
	TF(1-4)-Zif268	-
		
7F	Zif268	-
	Sp1C	-
	Sp1	-
	Sp1C-Zif268	-
	TF(1-4)-Zif268	1

**Table 3:** Applications and the relevant data in the database

**Database entries**	**Number/size of finger motifs**	**Framework** **Type/Class**	**Entry types**	**Output format**
Natural ZFP	2	C2H2	Positions	Web Interface
Natural ZFP	2	C2H2	Counts	Web Interface
Natural ZFP	3-13	CXXC and PHD	Positions	Web Interface
Natural ZFP	3-13	CXXC and PHD	Counts	Web Interface
Engineered ZFP	3	Sp1/C2H2	Positions	Web Interface
Engineered ZFP	3	Sp1/C2H2	Counts	Web Interface
Engineered ZFP	3, 4, 6	Zif 268/C2H2	Positions	Web Interface
Engineered ZFP	3, 4, 6	Zif 268/C2H2	Counts	Web Interface
Engineered ZFP	3, 6	Sp1c/C2H2	Positions	Web Interface
Engineered ZFP	3, 6	Sp1c/C2H2	Counts	Web Interface
Engineered ZFP	6	Sp1c-zif268/C2H2	Positions	Web Interface
Engineered ZFP	6	Sp1c-zif268/C2H2	Counts	Web Interface

#### Data access and generation

Data retrieval is an essential part of the database [10]. ZifBASE was implemented as a relational database. An interactive web interface was developed using server side scripts. The interface provides simple, advanced, and sequence-based searches.

## Utility and Discussion

ZifBASE is supported with a user-friendly designed web interface, so that user can easily get the desired information at any time. The index page of ZifBASE has three options and as follows:-

### ZifBASE search

**Figure 2:**
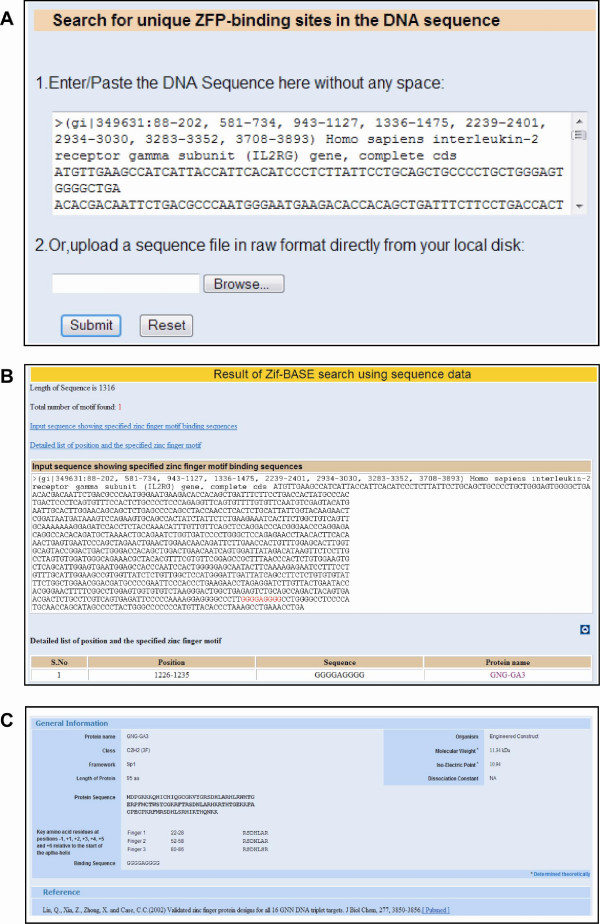
**ZifBASE search output**. Results of ZifBASE search using the sequence of *Homo sapiens *interleukin-2 receptor gamma subunit (IL2RG) gene. (A) Sequence input page (B) Result page showing the ZFP-binding site (in red color) predicted in the input sequence, along with a table showing the details of DNA sequence, position and ZFP name (linked to the respective entry in ZifBASE) (C) An engineered ZFP entry in ZifBASE showing seven positions (-1, +1, +2, +3, +4, +5 and +6 relative to the start of the α-helix) that makes most of the base contacts.

This option is mainly used to search the ZFPs and their target DNA-binding sites based on number of fingers (two, three, four, five, and above), framework (Zif 268, Sp1, Sp1C and Sp1C-Zif 268) and classes (C_2_H_2_, CXXC and PHD). ZifBASE also includes options to look for a specific ZFP or DNA binding site in the database and even for searching potential binding sites in a user-entered DNA sequence. They all differ in their features like number of fingers, type or class of fingers, recognition pattern in DNA and the source organism. The detailed list of engineered and natural ZFP can be obtained after ZifBASE search, which is connected into its output page by a hyperlink. The resulting page contains comprehensive information about ZFP with their features like protein name, protein sequences, recognition residues, position, framework, class, type, three-dimensional structure, DNA target site and references with PubMed citations (Figure 2). In addition, the molecular weight and isoelectric point of the each ZFP is calculated theoretically [18]. The interesting feature of ZifBASE-search is the integration of prediction results into the database entries. Figure 2 illustrates the type of information that is retrievable from ZifBASE and its subsequent usability. A specific *Homo sapiens *interleukin-2 receptor gamma subunit (IL2RG) gene sequence is keyed-in as input (Figure 2(A) and the result of ZifBASE-prediction tool shows that one ZFP binding site (in red color) predicted in the input sequence along with the table explaining the details about ZFP binding sequence, position and name of the ZFP. The predicted binding sequence has been hyper linked into database (Figure 2(B)). An engineered ZFP "GNG-GA3" recognizes this binding site with the help of three different fingers having the alpha-recognition helices as RSDHLAR (Position: 22-28), RSDNLAR (Position: 52-58) and RSDHLSR (Position: 80-86) as shown in Figure 2(C). In addition, information on the class, frame work, length, protein sequence, theoretical isoelectric point, molecular weight and PubMed reference are also displayed. The engineered construct (i.e. GNG-GA3) identified through ZifBASE can be used to target the interleukin-2 receptor gamma subunit gene sequence for targeted modification. In a similar manner, the user can predict the ZFP binding sites for targeted modification of plant and mammalian genomes. There is also a provision for the user to input any sequence to identify unique ZFP-binding sites in the DNA sequence. This is contingent upon the availability of the ZFP in ZifBASE that has been reported to bind to the user-given DNA sequence.

### Search for unique patterns in target DNA sequence

**Figure 3:**
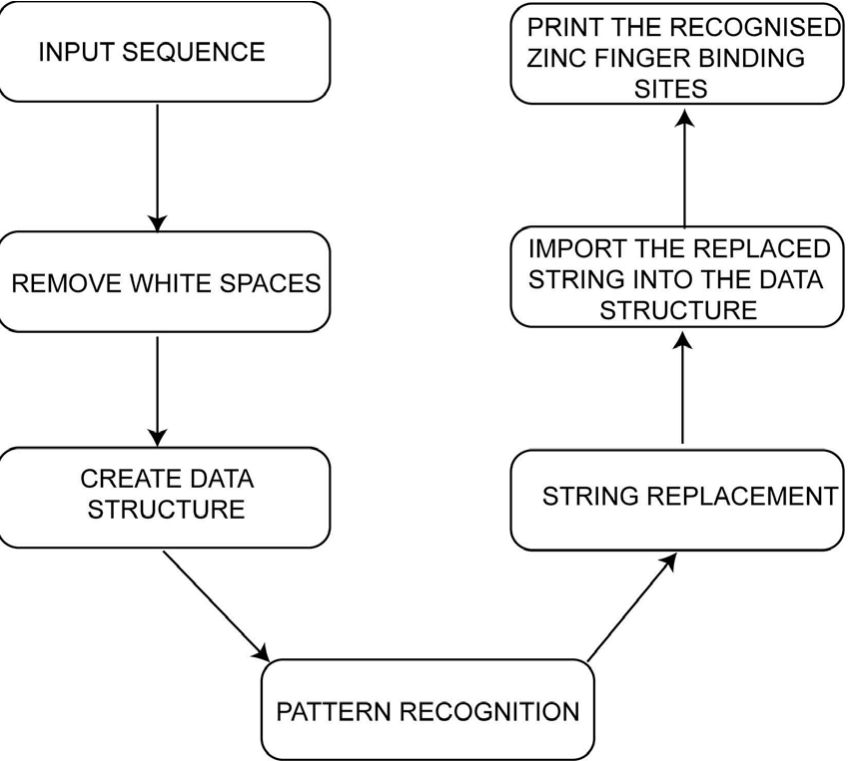
**The workflow of ZifBASE search tool**. Steps involved in identifying the potential zinc finger protein target sites from the given input query sequence.

This option is highly useful for molecular biologists who want to actively screen any complex DNA sequence to identify unique pattern in the sequence. The detailed workflow is shown in Figure 3. It uses two input parameters such as text area and file upload. The parameter 'file upload' is used to select a file from local disc, which contains sequence. Since ZF designs for GNN and ANN triplets are known, identifying these unique patterns will aid in design or evolving newer proteins. The search option can also handle a inverted repeat query sequence for zinc finger nuclease (ZFN) target sites [(NNPy)_4_.....(PyNN)_4 _or (NNN)_4_.....(NNN)_4 _separated by 2-5 bp spacer, where Py = T or C and N = G, A, T or C] [19]

### Subsequence search

This option allows the user to screen any gene sequence to identify unique gene-specific potential target sites for which ZFPs could be designed and/or selected by evolving DNA-binding affinity in zinc fingers.

## Conclusion

ZifBASE could be the most valuable resource of zinc finger proteins. This database was established based on the datasets of engineered and natural ZFP and it was curated manually. Importantly, the database provides sequence and structural features of natural ZFPs. Additional resources such as applications of zinc finger proteins, user guide and research publications are also provided. This database has dual significance: one is data retrieval of ZFP and its associated information and the other one is in search for potential target sites. UniProtKB, PDB, Protein Model Portal and ModBase references are given in resulting page of every search on natural ZFPs as additional information. It can greatly reduce the time in selection of unique gene-specific DNA target sites for which ZFPs could be designed or selected. ZifBASE was developed with two types of user in mind, bioinformaticians and molecular biologists. Bioinformaticians can identify conserved patterns of natural and engineered constructs of ZFPs and use this information to develop new prediction tools. Our database provides indispensable information of zinc finger proteins (containing multiple finger units) as it exists and not showing them separately as independent modules binding to a triplet each. The cross-strand interactions create synergy between neighboring finger motifs for the recognition of the 5'- base of each triplet-binding site. Individual finger motifs at a certain position in a constituent three-finger protein that binds to a target DNA triplet sequence, may not bind tightly to the same target if it has a different flanking finger motif. In addition, there will be a difference in binding affinity to the target sequence depending upon the suitable placing of individual finger motifs. Any attempt to build multi-finger peptides by joining individual fingers can now take the advantage of getting information on individual motifs, its position in the constituent ZFP along with its target triplet and flanking motifs. Details of multi-finger proteins with information on the strings of individual protein motif binding to a specific DNA triplet and their position are available in ZifBASE. The insight on cross-strand interactions will be highly useful for molecular biologists to address specificity of zinc fingers in an *in vivo *setting and designing or selecting them in the context of each other [20]. ZifBASE allows universal access to the database content and allows diverse queries sustaining many types of research utilizing zinc finger proteins. We trust that, this database will predominantly be precious for the rapidly rising number of molecular biologists involved in designing/evolving zinc finger proteins for targeting the genome locus of interest.

## Future directions

ZifBASE will be regularly updated with newly discovered and engineered ZFPs. ZifBASE will be developed further to include graphical representation of position of ZF motif based on a score function and prediction of ZFP DNA-binding site from the given amino acid sequence. A feature we plan to incorporate in the next version of ZifBASE is BLAST^ZF ^(BLAST for zinc finger nucleic acid and proteins) will be used to ensure the prediction results. In addition, the BLAST^ZF ^interface to find similar sequences to the most statistically significant sequences that are present in the ZifBASE will provide a way to expand ZifBASE in-depth as a first approach. The users can contribute newly found data that will be validated and incorporated in the database.

## Availability and requirements

The database, ZIF-Base, is now available at 

## Abbreviations

(ZFP): Zinc finger protein; (ZifBASE): zinc finger protein database;

## Competing interests

The authors declare that they have no competing interests.

## Authors' contributions

Corresponding author DS designed the method and framework for the project. MJ, JM, KC and DS wrote the manuscript. JM, SC and AP developed the MySQL database; MJ, KC and AP developed the web interface and related PHP scripts. All authors have read and approved the final manuscript.
